# Survey of knowledge, self-efficacy, and attitudes toward suicide prevention among nursing staff

**DOI:** 10.1186/s12909-023-04685-4

**Published:** 2023-09-22

**Authors:** Kuan Chin Huang, Shiow-Rong Jeang, Hsing-Ling Hsieh, Jing-Wen Chen, Ching-Hsien Yi, Yung-Chih Chiang, Han-Ping Wu

**Affiliations:** 1 Department of Nursing, Kaohsiung Veterans General Hospital, Kaohsiung, Taiwan; 2 Department of Nursing, Saint Paul’s Hospital, Taoyuan, Taiwan; 3 Department of Nursing, College of Medicine and Life Science, Chung Hwa University of Medical Technology, Tainan, Taiwan; 4 Extension School, Kaohsiung Medical University, Kaohsiung, Taiwan; 5 Department of Psychiatry, Kaohsiung Veterans General Hospital, No.386, Dazhong 1St Rd., Kaohsiung, 813414 Taiwan; 6 College of Medicine, Chang Gung University, Taoyuan, Taiwan; 7 Department of Pediatrics, Chiayi Chang-Gung Memorial Hospital, Chiayi County, No. 6, W. Sec. , Jiapu Rd., Puzi City, Taiwan

**Keywords:** Suicide prevention, Nursing staff, Knowledge, Self-efficacy, Attitude

## Abstract

**Objective:**

This study aimed to explore the knowledge, self-efficacy, and attitudes toward suicide prevention among nurses with different demographic characteristics.

**Methods:**

A cross-sectional descriptive design was adopted, and the study was conducted between August and September 2020. The content of the questionnaire included basic demographics, knowledge, self-efficacy, and attitudes toward suicide prevention. Correlation analysis was performed to determine nurses’ knowledge, self-efficacy, and attitudes toward suicide prevention.

**Results:**

The sample comprised 778 nursing staff from a medical center in southern Taiwan. The results showed that age, years of nursing experience, department type, education on suicide prevention, and care experience of nursing staff were associated with their knowledge, self-efficacy, and attitudes toward suicide prevention in general hospital patients. Younger and less experienced nurses demonstrated superior knowledge, self-efficacy, and attitudes toward suicide prevention. Nurses who received suicide-related education and training exhibited better self-efficacy and attitudes than those who did not. Nurses with care experience had better knowledge of suicide prevention than those without experience. Knowledge and self-efficacy in suicide prevention were both significantly and positively correlated with attitudes.

**Conclusion:**

Younger, less experienced, psychiatric nurses demonstrated superior knowledge, self-efficacy, and attitudes toward suicide prevention. Therefore, education on suicide prevention should be strengthened for older, experienced, and non-psychiatric nurses.

## Introduction

Suicide is a global public health concern. According to the World Health Organization (WHO) 2020 report, one person died of suicide every 40 s worldwide in 2019, and in 2016, suicide was the second leading cause of death among individuals aged 15 to 29 years [1]. Statistics released by the Ministry of Health and Welfare in Taiwan revealed that suicide ranked 11^th^ among all causes of death in 2020, with the number of suicide deaths totaling 3,656 and a crude suicide mortality rate per 100,000 population reaching 15.5 [2]. Each act of suicide has a major impact on the victim’s family, society, and nation [1].

A hospital is a place where one can find people with potential risk of suicide. A study conducted in Taiwan found that hospitalized patients in general hospitals had a higher risk of suicide mortality than the general population [3]. Another study in France found that psychiatric inpatients had a higher mortality rate than the general population and that the excess mortality rate was partially attributed to suicides [4]. A qualitative analysis study on suicide prevention among inpatients found that suicide peaks occurred shortly after admission and within one year of discharge [5]. The second annual report on suicide prevention in England pointed out that the first three months after hospital discharge was a high-risk period for suicide [6]. A systematic review and meta-analysis exploring suicide deaths showed that 3.7% of suicide deaths were inpatients, and 18.3% had contact with inpatient mental health services in the year before their death [7]. The emergency department is often the first medical care center where a suicide attempter is sent [8]. When patients attempt suicide, they may be assigned to psychiatric wards or non-psychiatric wards due to physical injuries [9]. Thus, hospital staff have access to patients at high risk of suicide. Among them, nursing staff provide primary care for these patients, are close to the patients, and have a better chance to evaluate and identify the signs of suicide [10, 11]. Thus, hospital nursing staff play a critical role in suicide prevention [12].

At the same time, some studies have found that emergency room nurses are more likely to reject, are unable to understand, and are likely to criticize suicide attempt cases, indicating that they only focus on physical care [13]. Other studies have shown that nursing staff in non-psychiatric units are more likely to exhibit negative attitudes toward patients with self-harming behaviors [14]. A study showed that the nurses’ negative attitudes may, to some extent, be a result of a lack of knowledge of suicide prevention [15]. The lack of knowledge of and negative attitudes toward suicide have a negative impact on health care and patient safety [16]. Also, the values and attitudes of nurses toward suicide may affect their willingness and ability to provide care [17–19]. A study investigating the prevalence of suicide misconceptions in Taiwan revealed that suicide misconceptions are prevalent among society [20]. Although some studies have examined nursing staff’s knowledge, attitude, and self-efficacy in suicide prevention [10, 15, 21], little is known about the correlation between nursing staff’s knowledge, attitude, and self-efficacy in suicide prevention and the factors that influence them. Therefore, this study aimed to explore the relationship between nurses’ knowledge, self-efficacy, attitudes toward suicide prevention and nurses’ demographic characteristics to provide a comprehensive understanding of the factors influencing suicide prevention in general hospital patient among hospital nursing staff.

## Methods

### Study design and participants

This cross-sectional descriptive study was conducted from August 17 to September 30, 2020. The participants were recruited through a total enumeration method, and included nursing staff assigned by the Nursing Department in a medical center in southern Taiwan; however, administrative nursing staff, specialist nurses, nursing staff at surgery rooms, recovery rooms, postpartum care homes, sick baby rooms, and nursing staff who were not involved in clinical care were excluded from the study. The nursing staff who met the criterion voluntarily participated in the study after reading the informed consent form.

### Research instruments

The demographic characteristics of nursing staff included the participants’ age, years of nursing experience, education, department type, level of experience in nursing clinical ladder programs, training received, and care experience for suicidal patients. The nursing clinical ladder program refers to a system for certifying nurses’ professional clinical abilities. It represents the nursing staff’s journey from being novices to clinical experts [22–24] and includes the lowest level N to the highest level N4, based on nurses’ work experience, completion of the nursing professional advancement program, and passing of all required performance evaluations.

The Nurses’ Knowledge of Suicide Prevention Questionnaire was compiled by the researchers based on relevant literature. The questionnaire contains nine items covering knowledge related to suicide prevention, including suicide epidemiology, suicide-related risk factor assessments, and suicide prevention [20, 25–28]. Correct responses were given one point, whereas incorrect responses or items marked as “Don’t Know” were scored zero, resulting in a scale ranging from zero to nine points. Experts suggest that the cut-off point should be set at 60 points, and a score above 60 represents a fair knowledge of suicide prevention. Moreover, higher scores indicated a better knowledge of suicide prevention.

The researchers also compiled the Nurses’ Self-Efficacy in Suicide Prevention Questionnaire which contained nine items and evaluated the participants’ confidence in their competence regarding the assessment of suicide cases, interview skills for suicide cases, integration of suicide prevention resources, and prevention of repeated suicide attempts. The questionnaire was scored on a five-point Likert scale, with one representing “strongly disagree” and five representing “strongly agree.” Higher scores indicated higher self-efficacy in suicide prevention. The content validity index (CVI) of these two questionnaires was determined by four experts—a physician, a psychologist, a social worker, and a university professor at the Department of Nursing—who were clinical practitioners in suicide prevention [29]. The CVI values of both questionnaires were 1, indicating good validity. The Test–Retest Reliability of the Nurses’ Knowledge of Suicide Prevention Questionnaire was acceptable (r = 0.761), and the internal consistency reliability of the Nurses’ Self-Efficacy in Suicide Prevention Questionnaire was very good (Cronbach’s α = 0.901).

The Attitudes to Suicide Prevention Scale was employed to measure nurses’ attitudes toward suicide prevention [30, 31]. The scale contains 14 items and is scored on a five-point Likert scale, with one representing “strongly disagree” and five representing “strongly agree,” resulting in a total score of 14–70 points. After accounting for reverse scoring, higher scores indicated more positive attitudes toward suicide prevention.

### Data collection

The researchers distributed the questionnaires to each ward of the participating hospital. The questionnaires were filled out and sent back to the researcher. The data from the questionnaires were collected, and personal information was coded and kept confidential. An overall analysis of the data was performed.

### Statistical analysis

Statistical analyses were performed using SPSS 20.0. The data were first presented as descriptive statistics, including the number of participants, percentage, mean, and standard deviation. For inferential statistics, one-way analysis of variance (ANOVA), analysis of covariance (ANCOVA), Scheffe and Games-Howell post-hoc tests, and Pearson product-moment correlation analysis were performed to understand the differences in knowledge, self-efficacy, and attitudes toward suicide prevention among nursing staff.

## Results

### Basic characteristics

A total of 849 questionnaires were distributed, of which 786 were recovered, and 778 were valid (91.6%). The mean age of the 778 participants was 33.59 ± 9.82 years and the mean years of nursing experience were 10.73 ± 9.39 years. The majority of participants were educated till the university level (87.7%) and achieved an N2 level (62.2%) in the nursing clinical ladder program. Among the different department types, general wards (57.2%) accounted for the highest number of participants, followed by intensive care wards (28.9%). A majority of nurses had attended one to three hours of suicide prevention training, and more than half (51.4%) had no experience in caring for suicidal patients over the previous year (Table 1).

**Table 1:** Basic characteristics of nursing staff (*N* = 778)

Variable	N	%	M ± SD	Minimum	Maximum
**Age**			33.59 ± 9.82	20	57
≤ 25 years	201	25.8			
26–35 years	291	37.4			
36–45 years	133	17.1			
> 46 years	153	19.7			
**Total years of nursing experience (in years)**			10.73 ± 9.39	0.1	33.8
≤ 1	77	9.9			
> 1–5	228	29.3			
> 5–10	160	20.6			
> 10–15	91	11.7			
> 15	222	28.5			
**Education level**					
Junior college	68	8.7			
University	682	87.7			
Master’s degree and above	28	3.6			
**Level of nursing clinical ladder program**					
N	109	14.0			
N1	64	8.2			
N2	484	62.2			
N3	74	9.5			
N4	47	6.0			
**Department type**					
Intensive care ward	225	28.9			
Emergency department	66	8.5			
General ward	465	59.8			
Psychiatric department	22	2.8			
**Attendance of suicide prevention training in the past year (in hours)**					
0 h	161	20.7			
1–3 h	569	73.1			
4–6 h	43	5.5			
≥ 7 h	5	0.6			
**Experience with caring for suicidal patients in the past year**					
0 patients	400	51.4			
1–5 patients	318	40.9			
6–10 patients	30	3.9			
≥ 11 patients	30	3.9			

### Knowledge of suicide prevention

Regarding knowledge scores for suicide prevention, the participants were asked a total of nine items related to the knowledge of suicide prevention, and the score (range: 0–9) of each questionnaire was divided by 9 and multiplied by 100 to obtain the total score (range: 0–100). The mean knowledge score was 68.85 ± 13.81, the mean number of correct responses was 6.20 items, and the rate of correct responses was 68.85%, of which the highest number of correct responses was eight and the lowest was zero. Further analysis of the individual scores for each item revealed that three items (items 1, 4, and 7) had lower-than-average rates of correct responses. Additional details are provided in Table 2.

**Table 2:** Scores and ranking of nursing staff’s knowledge of suicide prevention (*N* = 778)

Item	Rate of incorrect responses (%)	Rate of correct responses (%)	Note
1. More women than men die from suicide	64.3	35.7	^a^
2. Mental disorders and substance abuse are risk factors of suicide	8.5	91.5	
3. Discussing the suicidal thoughts of suicidal individuals will increase their risk of suicide	27.1	72.9	
4. Individuals who really want to commit suicide will not reveal relevant information or clues	48.7	51.3	^a^
5. Patients in remission from depression are at a lower risk of suicide	10.8	89.2	
6. The majority of individuals who reveal their suicidal thoughts or exhibit suicidal behaviors are asking for help through such actions	17.9	82.1	
7. Assessment of risk factors can help patients discover their potential and resilience to buffer them against suicidal crises	95.1	4.9	^a^
8. Suicide is usually caused by a single factor	5.0	95.0	
9. Most suicide survivors do not repeat suicide attempts	3.0	97.0	

^a^ indicates lower than average

### Self-efficacy in suicide prevention

Regarding nurses’ self-efficacy in suicide prevention, the mean scores for individual items ranged between 3.46 and 3.99, the total score ranged between 9 and 45, and the mean self-efficacy was 32.75 ± 4.49. Further analysis of the individual scores for each item revealed that items one, four, and eight had the lowest scores. Additional details are provided in Table 3.

**Table 3:** Scores and ranking of self-efficacy in suicide prevention in nursing staff (*N* = 778)

Item	M ± SD	Note
1. I have the ability to ask if a patient has suicidal intent	3.49 ± .72	^a^
2. I have the ability to discuss with other members of the medical team to determine if a patient is at risk of suicide	3.77 ± .63	
3. I have the ability to assess suicidal behavior	3.51 ± .69	
4. I can distinguish between patients at high and low risk of suicide	3.50 ± .68	^a^
5. I can guide patients to discuss their suicidal thoughts with a caring and empathetic attitude	3.77 ± .65	
6. I can provide emotional support and respond appropriately to the needs of patients at a high risk of suicide	3.69 ± .65	
7. I have the ability to perform suicide prevention measures, such as allocating beds close to the nurses’ station, taking note of dangerous objects in the environment that can be used for committing suicide, and asking family members to accompany the patient	3.99 ± .62	
8. I can help patients develop new coping strategies	3.46 ± .68	^a^
9. I have the ability to teach family members to identify suicide warning signs in patients	3.57 ± .67	

^a^indicates the three items with the lowest scores

### Attitudes toward suicide prevention

The total score of nurses’ attitudes toward suicide prevention ranged from 14 to 70, and the mean score was 47.69 ± 6.18. Items 6, 9, and 14 had the lowest scores. Additional details are provided in Table 4.

**Table 4:** Scores and ranking of attitudes toward suicide prevention in nursing staff (*N* = 778)

Item	M ± SD	Note
1. I dislike being asked about dealing with suicide	3.37 ± .92	
2. I believe suicide prevention is not my responsibility	4.10 ± .77	
3. I believe making more funds available to the appropriate health services would make no difference in the suicide rate	3.71 ± .87	
4. I believe that working with suicidal patients is rewarding	3.57 ± .76	
5. I believe that if someone is serious about committing suicide, they will not tell anyone	3.10 ± 1.14	
6. I feel defensive when people offer advice about suicide prevention	2.77 ± 1.03	^a^
7. I believe it is easy for people not involved in clinical practice to make judgments about suicide prevention	3.55 ± .78	
8. I believe if a person survives a suicide attempt, it was a ploy for attention	3.43 ± .98	
9. I believe people have the right to take their own lives	2.99 ± 1.08	^a^
10. As unemployment and poverty are the main causes of suicide, there is little I can do to prevent it	3.35 ± .92	
11. I do not feel comfortable assessing someone for suicide risk	3.48 ± .86	
12. I believe that measures for suicide prevention are a waste of resources, and they should be used elsewhere	4.04 ± .78	
13. I believe there is no way of knowing who is going to commit suicide	3.30 ± .77	
14. What proportion of suicide incidents do you consider preventable?	2.95 ± .75	^a^

^a^indicates the three items with the lowest scores

### Association between nurses’ demographic characteristics and knowledge, self-efficacy, and attitudes toward suicide prevention

Regarding knowledge of suicide prevention (shown in Table 5), nurses aged ≤ 25 years performed significantly better than those in the other three age groups (*p* < 0.05; Table 5). Nurses with ≤ 1 year of nursing experience performed better than those with > 5–10 and > 15 years of nursing experience (*p* < 0.05). Regarding the suicide prevention knowledge of nurses at different levels in the clinical ladder program, nurses at level N performed better than those at levels N2 and N4 (*p* < 0.05). Regarding department type, nurses working in the psychiatric department performed better than nurses working in the intensive care and general wards (*p* < 0.05). Nurses with experience in caring for one to five suicidal patients over the past year demonstrated better knowledge of suicide prevention than those who had no experience (*p* < 0.05).

**Table 5:** Association between demographic characteristics and nurses’ knowledge, self-efficacy, and attitudes toward suicide prevention (*N* = 778)

Item	M ± SD		
	Knowledge of suicide prevention	Self-efficacy in suicide prevention	Attitudes toward suicide prevention
**Age**			
≤ 25 years (A)	72.64 ± 12.86	33.27 ± 4.18	49.42 ± 6.47
26–35 years (B)	68.42 ± 13.19	33.03 ± 4.08	47.53 ± 6.22
36–45 years (C)	66.83 ± 13.26	31.96 ± 4.87	46.23 ± 6.24
> 46 years (D)	66.45 ± 15.63	32.19 ± 5.14	46.69 ± 6.18
F value	7.81***	3.49	8.7***
Group comparison	A > B*, C**, D***	-	A > B*, C***, D**
Years of nursing experience*^1^			
≤ 1 year (A)	73.74 ± 11.94	32.62 ± 4.60	49.96 ± 5.84
> 1–5 years (B)	70.96 ± 12.73	33.34 ± 3.80	49.74 ± 6.56
> 5–10 years (C)	67.29 ± 13.78	33.11 ± 4.24	47.31 ± 5.93
> 10–15 years (D)	66.40 ± 12.15	32.34 ± 4.97	45.58 ± 6.98
> 15 years (E)	66.72 ± 15.44	32.09 ± 4.97	46.97 ± 5.28
F value	5.97***	2.68*	8.09***
Group comparison	A > C*, E**: B > E*	B > E*	A > C*, D***, E** B > D**, E*
**Education level**			
Junior college	69.93 ± 13.85	32.97 ± 5.17	47.47 ± 6.92
University	68.87 ± 13.64	32.70 ± 4.43	47.72 ± 6.13
Master’s degree and above	65.87 ± 17.61	33.39 ± 4.43	47.64 ± 5.82
F value	.86	.41	.05
Group comparison	-	-	-
**Nursing clinical ladder program**			
N (A)	72.68 ± 12.05	33.10 ± 4.28	49.13 ± 6.46
N1 (B)	72.22 ± 14.28	33.48 ± 4.12	49.45 ± 6.12
N2 (C)	67.98 ± 13.98	32.72 ± 4.34	47.25 ± 6.19
N3 (D)	69.07 ± 11.38	32.34 ± 5.20	46.72 ± 5.86
N4 (E)	64.07 ± 16.25	31.87 ± 5.62	48.09 ± 5.12
F value	5.05**	1.21	3.97
Group comparison	A > C*, E*: B > E*	-	-
**Department type**			
Intensive care ward (A)	67.01 ± 14.86	35.56 ± 4.78	47.00 ± 6.33
Emergency department (B)	69.53 ± 12.53	32.00 ± 3.95	45.73 ± 5.86
General ward (C)	68.20 ± 13.28	32.67 ± 4.25	48.08 ± 6.03
Psychiatric department (D)	78.28 ± 11.10	38.55 ± 4.19	52.36 ± 5.69
F value	4.97**	13.64***	8.20***
Group comparison	D > A**, C*	D > A**, B***, C***	C > B***; D > A**, B***, C*
Hours of suicide prevention training attended in the past year*^2^			
0 h (A)	68.32 ± 15.17	31.25 ± 4.24	46.48 ± 6.18
1–3 h (B)	68.72 ± 13.64	33.03 ± 4.43	47.97 ± 6.11
4–6 h (C)	71.32 ± 10.35	34.37 ± 4.83	48.19 ± 6.65
≥ 7 h (D)	80.00 ± 9.30	34.20 ± 6.38	50.40 ± 7.02
F value	1.64	9.02***	2.90
Group comparison	-	B > A***; C > A***	-
Number of suicidal patients cared for in the past year*^3^			
0 (A)	67.42 ± 14.56	32.43 ± 4.45	47.41 ± 6.22
1–5 (B)	70.34 ± 12.46	32.92 ± 4.33	48.25 ± 5.81
6–10 (C)	70.25 ± 13.57	32.77 ± 4.68	45.35 ± 6.80
≥ 11 (D)	70.74 ± 16.11	35.07 ± 5.84	48.00 ± 8.09
F value	2.97*	3.50*	2.65
Group comparison	B > A*	D > A*	-

Statistic method: One-way analysis of variance (ANOVA), Scheffe and Games-Howell post-hoc tests, and Pearson product-moment correlation analysis

^*^p < .05. **p < .01. ***p < *.001*

^*1*2*3^ showed significant low positive correlations

Regarding self-efficacy in suicide prevention, no significant differences were identified among nurses of different ages, education, and levels in the nursing clinical ladder program. Nurses with > 1–5 years of nursing experience performed better than those with > 15 years of nursing experience (*p* < 0.05). Among the different department types, nurses working in the psychiatric department performed better than those working in the intensive care wards, emergency departments, and general wards (*p* < 0.001). Nurses who attended one to three hours and four to six hours of suicide prevention training in the past year performed better than those who did not attend such courses (*p* < 0.01). Nurses who cared for ≥ 11 suicidal patients in the past year demonstrated superior self-efficacy in suicide prevention compared to those who cared for no suicidal patients (*p* < 0.05).

Regarding attitude toward suicide prevention, nurses aged ≤ 25 years with ≤ 1 year of nursing experience scored the highest (*p* < 0.05; Table 5). Among the different department types, nurses working in the psychiatric department scored higher than those working in the intensive care wards, emergency departments, and general wards (*p* < 0.05), whereas nurses working in general wards performed better than those working in the emergency departments (*p* < 0.05).

### Relationship between knowledge, self-efficacy, and attitudes toward suicide prevention

Nurses’ knowledge of and self-efficacy in suicide prevention showed significant positive correlations with attitudes toward suicide prevention. Knowledge of suicide prevention was also positively correlated with self-efficacy in suicide prevention; however, the correlation was not statistically significant (Table 6).

**Table 6:** Correlation analysis among knowledge, self-efficacy, and attitudes toward suicide prevention (*N* = 778)

	Knowledge of suicide prevention	Attitudes toward suicide prevention
Attitudes toward suicide prevention	.353***	-
Self-efficacy in suicide prevention	.299	.297***

Statistic method: Pearson product-moment correlation analysis

^*^p < .05. **p < .01. ***p < *.001*

## Discussion

This study evaluates the knowledge, self-efficacy, and attitude toward suicide prevention of nursing staff in a general hospital and analyzes the influence of related factors. Overall, the mean knowledge score on suicide prevention exceeded 60 (full score 100), and the mean score for self-efficacy and attitude were both higher than the midpoint, indicating that the nursing staff had a fair knowledge, self-efficacy and attitude on suicide prevention. Regarding knowledge scores toward suicide precaution, three items had a score lower than 60: “Assessment of risk factors can help patients discover their potential and resilience to buffer them against suicidal crises,” “More women than men die from suicide,” and “Individuals who are serious about committing suicide will not reveal any information or clues.” This suggests slight deficiencies or misconceptions in nursing staff’s knowledge regarding suicide-related assessments. Regarding attitudes toward suicide prevention, the three items that scored lower than the midpoint were: “I feel defensive when people offer advice about suicide prevention,” “What proportion of suicide incidents do you consider preventable?” and “I believe people have the right to take their own lives.” These findings demonstrate that nursing staff had an inadequate grasp of suicide prevention and found it difficult to discuss the right to autonomy over life and suicide. Prior studies demonstrated that suicide prevention training can enhance knowledge of suicide prevention and risk assessment [32] while improving nurses’ self-efficacy in caring for patients with suicidal ideations [33]. However, most nursing professionals felt that they had not received adequate suicide prevention training [10, 34].

Based on this study’s findings, nursing staff’s knowledge, self-efficacy, and attitudes toward suicide prevention were affected by demographic characteristics like age, years of nursing experience, department type, education and training received, and care experience. Specifically, younger and less experienced nurses showed better knowledge of, self-efficacy in, and attitudes toward suicide prevention, whereas older and more experienced nurses demonstrated poorer knowledge of, self-efficacy in, and attitudes toward suicide prevention. Previous studies found that younger staff demonstrated better skills and attitudes toward suicide prevention than older staff because younger staff were more receptive to new ideas, more empathetic toward suicidal patients, and more passionate about their work [15, 18]. This study’s results also showed that nurses at the N4 level of the clinical ladder program (6%, *n* = 47), with > 15 years of nursing experience (28.5%, *n* = 222) performed the poorest regarding knowledge, self-efficacy, and attitudes toward suicide prevention. Since caring for suicidal patients requires more empathy and perceptiveness, nurses who are older, at higher levels of the clinical ladder program, and more experienced should be more attentive of suicide prevention compared to younger and less experienced nurses [15, 21]. Some studies have shown that nursing staff with higher levels of education have more positive attitudes toward suicide prevention [15, 21] while others have found that education does not affect nurses’ attitudes [14]. In this study, no significant difference was identified among nurses with different education levels, which may have been due to the fact that the majority of participants in this study were educated till the university level (87.7%, *n* = 682), while the number of participants with graduate degrees (3.6%, *n* = 28) was relatively small.

Given that the attitudes of nursing staff can affect their nursing behavior, staff with negative attitudes toward suicide prevention may cause potential harm to their patients. In this study, 79.3% of the nursing staff had attended less than one hour of suicide prevention-related courses in the past year, and the knowledge score for suicide prevention exceeded 60 (total score: 100), while the scores for self-efficacy and attitude were both higher than the midpoint (midpoint: 3). This indicated that the participants had adequate knowledge, confidence, and willingness to take care of suicidal patients. Furthermore, this study’s findings also indicated that nurses with experience caring for one to five suicidal patients demonstrated better knowledge of suicide prevention than those without such experience, while nurses with experience of caring for more than 11 suicidal patients had better self-efficacy in suicide prevention than those without experience. This finding indicates that care experience may affect nurses’ knowledge of and confidence in suicide prevention. Regarding suicide-related training, although there were no statistically significant differences among the groups, the results showed that nurses who attended more hours of training had higher mean scores, implying that attending suicide-related training contributed to an improvement in knowledge to some extent. Moreover, nurses who attended one to three hours and four to six hours of training had significantly higher self-efficacy than those who did not. Therefore, education and training improved nurses’ knowledge of suicide prevention and also increased their level of self-efficacy in suicide prevention. However, there is a positive correlation between years of nursing experience, hours of suicide prevention training, and the number of suicidal patients cared for. Thus, the correlation of these variables and their impact on suicide prevention knowledge, self-efficacy, and attitudes could be complex and difficult to clarify based on the analysis provided in this study. In addition, the knowledge and self-efficacy of nursing staff regarding suicide prevention were positively correlated with their attitudes. This implies that enhancing either knowledge or self-efficacy in suicide prevention can contribute to improving attitudes toward suicide prevention. Previous studies on primary caregivers or nursing staff indicated that the ability to prevent suicides is positively correlated with attitudes toward suicide prevention [17, 18], while another study demonstrated that knowledge of and confidence in suicide prevention had an impact on attitudes and could affect patient care outcomes [35]. These results correspond with this study’s findings.

Among the different department types, psychiatric nurses exhibited superior knowledge, self-efficacy, and attitudes toward suicide prevention compared to nurses in other departments. Furthermore, Clua-García et al. [36] exhibited that psychiatric nurses receive better training on therapeutic relationships, continuing education related to suicide training, and more mutual support from team members compared to the nursing staff of other departments. Consequently, they experienced fewer negative emotions when dealing with suicide attempts and could provide more effective care to suicidal patients [14]. However, it is worth noting that emergency and psychiatric nurses showed no significant difference in their knowledge of suicide prevention but differed significantly regarding self-efficacy in and attitudes toward suicide prevention. Moreover, nurses working in general wards performed significantly better than emergency nurses concerning attitudes toward suicide prevention. A study conducted in the United States on 224 patients who sought health care within 12 months of a suicide attempt revealed that the previous medical visit of 42.4% of the patients was to the emergency department [37]. This highlights the importance of emergency nurses in suicide prevention. Furthermore, several studies have demonstrated that nursing staff who lack understanding and acceptance of suicide attempt cases and believe that they are inadequately trained in suicide prevention may feel fearful of discussing suicide with their patients or lack sufficient sensitivity, which can prevent them from identifying and providing timely assistance to patients at risk of suicide. Therefore, the provision of suicide-related training courses can improve the self-efficacy and attitudes of nursing staff toward suicide prevention [10, 13, 34, 38–40].

One of the limitations of our study is that we only investigated the nursing staff of a single institution, and therefore, it might not be possible to generalize the results of study. However, this study involved a relatively large number of cases with a high participation rate, which should be representative of the state of nursing staff in a general hospital. Another limitation is that this is a cross-sectional study based on self-administered structured questionnaires, and thus, it did not explore in depth the factors influencing nursing staff’s knowledge, self-efficacy, and attitudes toward suicide precaution and their causal relationship. Finally, the knowledge and self-efficacy items were developed by the researchers. This was because no valid and reliable Chinese version of the Nursing’s Knowledge on of Suicide Prevention Questionnaire and Nursing’s Self-efficacy in Suicide Prevention Questionnaire was available when this research was designed in 2019. Nevertheless, we conducted an analysis of reliability and validity of these two questionnaires before executing this study and the results were acceptable. Furthermore, this is one of the few studies so far that simultaneously analyzes the knowledge, self-efficacy, and attitudes of nursing staff toward suicide prevention. The preliminary results of this study could be considered a reference for subsequent researchers.

## Conclusions

Younger and less experienced nurses demonstrated superior knowledge, self-efficacy, and attitudes toward suicide prevention. Nurses who received suicide-related education and training exhibited better self-efficacy and attitudes toward suicide prevention than those who did not. Knowledge and self-efficacy in suicide prevention were both significantly positively correlated with attitudes, which may affect care quality and is thus critical to patient safety. It is suggested that suicide prevention courses should be included in the training of nursing staff, including older and more experienced nurses. Further research is warranted to explore broadly and deeply the factors influencing nursing staff’s knowledge, self-efficacy, and attitudes toward suicide precaution, and the causal relationship between them.

## Data Availability

The raw data supporting the conclusions of this article will be made available on request to the corresponding authors.

## References

[1] World Health Organization. Suicide; 2020. https://www.who.int/zh/news-room/fact-sheets/detail/suicide. Accessed 6 March 2022.

[2] Ministry of Health and Welfare. Suicide statistics;2021. https://www.mohw.gov.tw/dl-70945-f9e765cd-2149-43ac-ba8e-5ceceb8b315c.html. Accessed 6 March 2022.

[3] Tseng MC, Cheng IC, Hu FC (2011). Standardized mortality ratio of inpatient suicide in a general hospital. J Formos Med Assoc.

[4] Casadebaig F, Quemada N (1989). Mortality in psychiatric inpatients.

[5] Sakinofsky I (2014). Preventing suicide among inpatients. Can J Psychiatry.

[6] UK GOV: Preventing suicide in England: Two years on Second annual report on the cross-government outcomes strategy to save lives. http://www.tinyurl.com/mlxoalk. Accessed 6 March 2022;2015.

[7] Walby FA, Myhre M, Kildahl AT (2018). Contact with mental health services prior to suicide: a systematic review and meta-analysis. Psychiatr Serv.

[8] Owens PL, McDermott KW, Lipari RN, Hambrick MM. Emergency Department Visits Related to Suicidal Ideation or Suicide Attempt, 2008–2017. In: Healthcare Cost and Utilization Project (HCUP) Statistical Briefs*.* edn. Rockville (MD): Agency for Healthcare Research and Quality (US); 2006. 33074641

[9] Kim HJ, Lee DH (2021). Predictive factors for the medical hospitalisation of patients who visited the emergency department with suicide attempt. BMC Psychiatry.

[10] Bolster C, Holliday C, Oneal G, Shaw M (2015). Suicide assessment and nurses: what does the evidence show?. Online J Issues Nurs.

[11] De Silva A, Dawson AH, Gawarammana IB, Tennakoon S, Rajapakse T (2018). Study protocol: a pilot randomized controlled trial to evaluate the acceptability and feasibility of a counseling intervention, delivered by nurses, for those who have attempted self-poisoning in Sri Lanka. Pilot and feasibility studies.

[12] Ghebrehiwet T, Barrett T (2007). Nurses and mental health services in developing countries. Lancet.

[13] Vedana KGG, Magrini DF, Miasso AI, Zanetti ACG, de Souza J, Borges TL (2017). Emergency nursing experiences in assisting people with suicidal behavior: a grounded theory study. Arch Psychiatr Nurs.

[14] Pintar Babič M, Bregar B, Drobnič Radobuljac M (2020). The attitudes and feelings of mental health nurses towards adolescents and young adults with nonsuicidal self-injuring behaviors. Child Adolesc Psychiatry Ment Health.

[15] Ouzouni C, Nakakis K (2013). Nurses' attitudes towards attempted suicide. Health Sci J.

[16] Neville K, Roan NM. Suicide in hospitalized medical-surgical patients: exploring nurses' attitudes. J Psychosoc Nurs Ment Health Serv. 2013; 51(1):35–43; quiz 44–35. 10.3928/02793695-20121204-01 23231401

[17] Chiang CY, Lu CY, Lin YH, Lin HY, Sun FK (2015). Caring stress, suicidal attitude and suicide care ability among family caregivers of suicidal individuals: a path analysis. J Psychiatr Ment Health Nurs.

[18] Lygnugaryte-Griksiene A, Leskauskas D, Jasinskas N, Masiukiene A (2017). Factors influencing the suicide intervention skills of emergency medical services providers. Med Educ Online.

[19] Saigle VH. Suicide and ethics: a review of practical issues that arise in clinical and research settings and their neglect in academia. (Master's thesis). http://digitool.library.mcgill.ca/webclient/StreamGate?folder_id=0&dvs=1505547427473~79. Accessed 6 2022; 2016.

[20] Wang YT, Chang SS, Chi YC, Chien-Chang Wu K, Chen YY. Suicide misconceptions and attitudes toward suicide prevention measures in Taiwan. Crisis. 2022. 10.1027/0227-5910/a000893. 10.1027/0227-5910/a000893 36444884

[21] Sun FK, Long A, Boore J (2007). The attitudes of casualty nurses in Taiwan to patients who have attempted suicide. J Clin Nurs.

[22] Burket TL, Felmlee M, Greider PJ, Hippensteel DM, Rohrer EA, Shay ML (2010). Clinical ladder program evolution: journey from novice to expert to enhancing outcomes. J Contin Educ Nurs.

[23] Ko YK, Yu S (2014). Clinical ladder program implementation: a project guide. J Nurs Adm.

[24] Pierson MA, Liggett C, Moore KS (2010). Twenty years of experience with a clinical ladder: a tool for professional growth, evidence-based practice, recruitment, and retention. J Contin Educ Nurs.

[25] Bachmann S (2018). Epidemiology of Suicide and the Psychiatric Perspective. Int J Environ Res Public Health.

[26] Liao S-C, Lee M-B, Lung F (2015). Wu C-y, Chang C-M: Suicide prevention in Taiwan: A ten-year review. Taiwan J Public Health.

[27] Wasserman D, Rihmer Z, Rujescu D, Sarchiapone M, Sokolowski M, Titelman D, Zalsman G, Zemishlany Z, Carli V (2012). The European Psychiatric Association (EPA) guidance on suicide treatment and prevention. Neuropsychopharmacol Hung.

[28] Berardelli I, Aguglia A, Cassioli E, Bersani FS, Longo L, Luciano M, Minichino A, Santambrogio J, Solmi M, Rossi R, et al. Suicide-related knowledge among Italian early career psychiatrists and trainees: results from a cross-sectional survey. Brain Sci. 2022; 12(12). 10.3390/brainsci12121619 PMC9776389 36552079

[29] Yusoff MSB (2019). ABC of content validation and content validity index calculation. Education in Medicine Journal.

[30] Appleby L, Morriss R, Gask L, Roland M, Perry B, Lewis A, Battersby L, Colbert N, Green G, Amos T (2000). An educational intervention for front-line health professionals in the assessment and management of suicidal patients (The STORM Project). Psychol Med.

[31] Chang CM, Lai TJ, Chou MC, MC L. The Experiences, knowledge, attitudes and confidence of general practitioners in suicide prevention. Taiwanese J Psychiatry. 2006, 20(2):134-144

[32] Elzinga E, de Kruif A, de Beurs DP, Beekman ATF, Franx G, Gilissen R (2020). Engaging primary care professionals in suicide prevention: a qualitative study. PLoS One.

[33] Kerr S, Martin C, Fleming M (2018). Preventing suicide; nurse education and the occluded issue of gender. Nurse Educ Pract.

[34] Giacchero Vedana KG, Magrini DF, Zanetti ACG, Miasso AI, Borges TL, Dos Santos MA (2017). Attitudes towards suicidal behaviour and associated factors among nursing professionals: a quantitative study. J Psychiatr Ment Health Nurs.

[35] Boukouvalas E, El-Den S, Murphy AL, Salvador-Carulla L, O’Reilly CL (2020). Exploring health care professionals’ knowledge of, attitudes towards, and confidence in caring for people at risk of suicide: a systematic review. Arch Suicide Res.

[36] Clua-García R, Casanova-Garrigós G, Moreno-Poyato AR (2021). Suicide care from the nursing perspective: a meta-synthesis of qualitative studies. J Adv Nurs.

[37] Stuck AR, Wilson MP, Chalmers CE, Lucas J, Sarkin A, Choi K, Center K (2017). Health care usage and suicide risk screening within 1 year of suicide death. J Emerg Med.

[38] Blair EW, Chhabra J, Belonick C, Tackett M (2018). Non-psychiatric nurses' perceived self-efficacy after an educational intervention on suicide prevention and care. J Psychosoc Nurs Ment Health Serv.

[39] Lerchenfeldt S, Kamel-ElSayed S, Patino G, Thomas DM, Wagner J (2020). Suicide assessment and management team-based learning module. MedEdPORTAL.

[40] Maina R, Bukusi D, Kumar M (2019). Suicide prevention by emergency nurses: perceived self-efficacy in assessment, management and referral at Kenyatta National Hospital in Kenya. Ann Gen Psychiatry.

